# Phylogenetic Signal, Root Morphology, Mycorrhizal Type, and Macroinvertebrate Exclusion: Exploring Wood Decomposition in Soils Conditioned by 13 Temperate Tree Species

**DOI:** 10.3390/f13040536

**Published:** 2022-03-30

**Authors:** Rondy J. Malik, Mary Ann V. Bruns, Terrence H. Bell, David M. Eissenstat

**Affiliations:** 1 Kansas Biological Survey, The University of Kansas, 2101 Constant Ave, Lawrence, KS 66045, USA; 2 Department of Ecosystem Science and Management, Penn State University, University Park, PA 16802, USA; 3 Department of Plant Pathology and Environmental Microbiology, Penn State University, University Park, PA 16802, USA

**Keywords:** mycorrhizae, root morphology, woody plants, decomposition, phylogenetic signals

## Abstract

Woodlands are pivotal to carbon stocks, but the process of cycling C is slow and may be most effective in the biodiverse root zone. How the root zone impacts plants has been widely examined over the past few decades, but the role of the root zone in decomposition is understudied. Here, we examined how mycorrhizal association and macroinvertebrate activity influences wood decomposition across diverse tree species. Within the root zone of six predominantly arbuscular mycorrhizal (AM) (*Acer negundo, Acer saccharum, Prunus serotina, Juglans nigra, Sassafras albidum,* and *Liriodendron tulipfera*) and seven predominantly ectomycorrhizal (EM) tree species (*Carya glabra, Quercus alba, Quercus rubra, Betula alleghaniensis, Picea rubens, Pinus virginiana,* and *Pinus strobus*), woody litter was buried for 13 months. Macroinvertebrate access to woody substrate was either prevented or not using 0.22 mm mesh in a common garden site in central Pennsylvania. Decomposition was assessed as proportionate mass loss, as explained by root diameter, phylogenetic signal, mycorrhizal type, canopy tree trait, or macroinvertebrate exclusion. Macroinvertebrate exclusion significantly increased wood decomposition by 5.9%, while mycorrhizal type did not affect wood decomposition, nor did canopy traits (i.e., broad leaves versus pine needles). Interestingly, there was a phylogenetic signal for wood decomposition. Local indicators for phylogenetic associations (LIPA) determined high values of sensitivity value in *Pinus* and *Picea* genera, while *Carya, Juglans, Betula,* and *Prunus* yielded low values of sensitivity. Phylogenetic signals went undetected for tree root morphology. Despite this, roots greater than 0.35 mm significantly increased woody litter decomposition by 8%. In conclusion, the findings of this study suggest trees with larger root diameters can accelerate C cycling, as can trees associated with certain phylogenetic clades. In addition, root zone macroinvertebrates can potentially limit woody C cycling, while mycorrhizal type does not play a significant role.

## 1. Introduction

Woody plants are quintessential to carbon storage and net primary productivity [1,2]. These rigid organisms inhabit 30% of Earth’s landmass and contain 50% of the carbon that makes up the aboveground terrestrial biosphere [3,4]. In addition to their impact on C flux, woody plants can shape the pedosphere [5] through root recruitment of mycorrhizal fungi, recycling of organic material, and litter deposition [6,7]. Particularly, plant material can be fragmented by natural events (e.g., freeze–thaw events, windstorms, and animal activity), as well as deposited into soil communities from plant standing mass. Coarse and fine wood debris can make up anywhere from 1 to 25% of the forest floor [8-10], as the brown food web is supplemented by rotten logs, snags and stumps, which can provide resources for seed germination [11], as well as habitats for invertebrates and microbes. Select soil microbes have a primary role in wood degradation and are key components of SOM formation and decomposition [12]. Yet, soil macroinvertebrates (>2 mm) may have an equally important role as microbial decomposers [13], but macroinvertebrate function is often context dependent and can at times be disruptive (e.g., burrowing, predation, and ecosystem engineering) to microbial function.

Determining ways in which diverse root zones may impact soil fauna is essential to understanding brown food web processes. Many organisms are involved in mineralizing wood: ~20–30 percent of forest arthropods, including insects, are either wood dependent (i.e., saproxylic) or opportunistically utilize wood [14,15]. Wood can be broken down through modes of tunneling and nesting [16], while some invertebrates rely on hindgut microbes to metabolize wood [17,18]. Lumbricida (i.e., earthworms) and collembola (i.e., springtails) harbor *Bacillus* spp. in their intestinal systems, which plays a role in the degradation of chitin and lignocellulose [17]. Radiolabeling studies have found invertebrates to consume both mycorrhizae and saprotrophic fungi [19], perhaps suggesting that macroinvertebrates can harness microorganisms involved in C exchange and cycling. Invertebrate activity, including the communition of wood debris and burrowing activity, can increase wood surface area, potentially leading to increased decomposition through mycelium contact with wood surfaces. Invertebrates may also slow down decomposition, as collembola (i.e., *Folsomia Candida*) activity has been reported to sever and disrupt mycelial cords from connecting with wood substrate [20], which can slow C cycling. This may be pertinent to recent studies that suggest hyphal extension to be an important predictor of wood decomposition [21].

Wood is decomposed by a limited number of invertebrate and fungal classes [15,22,23]. Decomposer efficacy may also be influenced by foliar litter input [24-26], as minerals can differentially accumulate in foliar tissue prior to being introduced into soils [27]. Differences in pine versus broad leaf tissue may differentially alter soil chemistry (i.e., pH) [28], as aging pine needles can lead to an increase in Mn^2+^ oxidation state (i.e., Mn^3+^ and Mn^4+^), which negatively corresponds with C: N [29]. Such changes in litter chemistry may directly affect decomposers [30], as white, brown, and soft rot presence in sapwood corresponds to wood pH [31]. White and brown rot fungi can decompose wood through enzymatic activity or Fenton redox chemistry [32-35].

Aside from free-living decomposers, root-associated fungi may also play a role in decomposition, especially in the context of the root zone. Some root-partnering fungi, termed ectomycorrhizal (EM) fungi, have been reported to degrade cellulose through Fenton redox chemistry and hydroxyl radical attacks [36,37]. This suggests that the life history of EM fungi spans a biotroph–saprotroph continuum [38], but this characterization may depend on life history, evolutionary divergence, and the retention/expression of lignocellulolytic genes that are localized in mycelial networks [39]. In contrast, arbuscular mycorrhizal (AM) fungi are not known for lignocellulolytic capabilities, but still, AM fungi can increase water-stable aggregates [40,41] and make the soil environment conducive toward decomposers. Interestingly, fine roots can reduce soil moisture (i.e., drying effect), which may be antagonistic toward decomposers [42]. Thus, root morphology, or variation in root diameter, may help shape root zone decomposition, as previous studies found correlations with root diameter and soil organic matter respiration [43,44]. Moreover, root-zone-mediated decomposition may also be autocorrelated with phylogenetic signals or links between phylogeny and continuous trait values [45]. For example, decomposition in hardwood tree soils (e.g., black cherry) may differ from softwoods (e.g., pines), but in a manner that is not to be confused with phylogenetic conservatism [46].

To date, a myriad of studies has increased our understanding of wood decomposition [47-51], but the effect of the soil-mediated root zone is unclear. In addition to that, it is unclear how macroinvertebrates are differentially influenced by the root zone of EM-versus AM-associated trees, as well as trees of varying root diameter and phylogenetic distance. In this study, we sought to unravel how soils conditioned by diverse tree species can affect the decomposition of woody litter. Here, mycorrhizal type, phylogenetic distance, root diameter, litter quality (canopy trait), and macroinvertebrates were assessed. To address this knowledge gap, we sought to determine how mycorrhizal type interacts with macroinvertebrates to influence decomposition. To limit the influence of environmental heterogeneity, we made use of a common garden forest by manipulating the presence (+) or absence (−) of macroinvertebrates > 0.22 mm on added woody material in the root zone of six AM and seven EM trees. Under these conditions, wood was allowed to decompose for 13 months in the root zone of 13 tree species. We hypothesized that: (1) the exclusion of macroinvertebrates (>0.22-mm) would enhance decomposition due to less negative interactions on microbes; (2) root diameter would be a good predictor of decomposition, and there might be a phylogenetic signal for this trait. Additionally, (3) differences in mycorrhizal association would lead to differences in wood decomposition rates.

## 2. Materials and Methods

### 2.1. Study Site

This study was conducted within a common garden forest in central Pennsylvania, USA (40° 42’ N, 77°57’ W). From 1981 to 2010, annual rainfall in this area has been ~1006 mm, and the average annual high temperature is recorded as 15 °C and average annual low temperature is recorded as 5 °C (National Climatic Data Center [52]). The research site rests on continuously flat semi-active mesic Typic Hapludalfs soil composed of silt loam (Hagerstown series, 0–3 percent slope), with an approximate pH of 5.48 [53]. The previous land use at this site was grassy hay-field (Cheng et al., 2016), prior to becoming a research site in the mid-1990s. This site features tree species occurring in conspecific monocultures (i.e., conspecific blocks), which is a stand of 6 trees of the same species separated belowground from other species by plastic barriers to approximately a 1 m depth. The trees are over 20 years old, and prior research at this site has described root morphology and mycorrhizal root colonization levels [54,55].

### 2.2. Study System

Macroinvertebrates can be characterized on a size-class basis, as size-class delineation does not take away from the essential role of invertebrates involved in brown food webs, resource redistribution, and decomposition. Soil macroinvertebrates, ranging from earthworms, soil-dwelling insects, myriapods, isopods, and Staphylinids, are of a size class larger than 2 mm [56,57] and differ from the meso fauna that are between 0.1 mm and 0.2 mm [13,58,59]. The manipulation of invertebrate presence, absence, or density can require laborious sterilization or inoculation in a controlled setting [60], but as it relates to this study, polyester mesh (0.22 mm) was used to restrict macroinvertebrates from interacting with decomposing wood substrate placed in the root zone of 6 AM and 7 EM trees.

The impact of the root zone of 6 AM (*Acer negundo, Acer saccharum, Prunus serotina, Juglans nigra, Sassafras albidum,* and *Liriodendron tulipfera*) and 7 EM trees (*Carya glabra, Quercus alba, Quercus rubra, Betula alleghaniensis, Picea rubens, Pinus virginiana,* and *Pinus strobus*) (Table 1) on wood decomposition was compared. AM and EM trees are known to differentially impact underlying soil and microorganisms, and at our site, AM-associated trees generally had increased soil pH and available N [61].

### 2.3. Experimental Design

**Figure 1.**
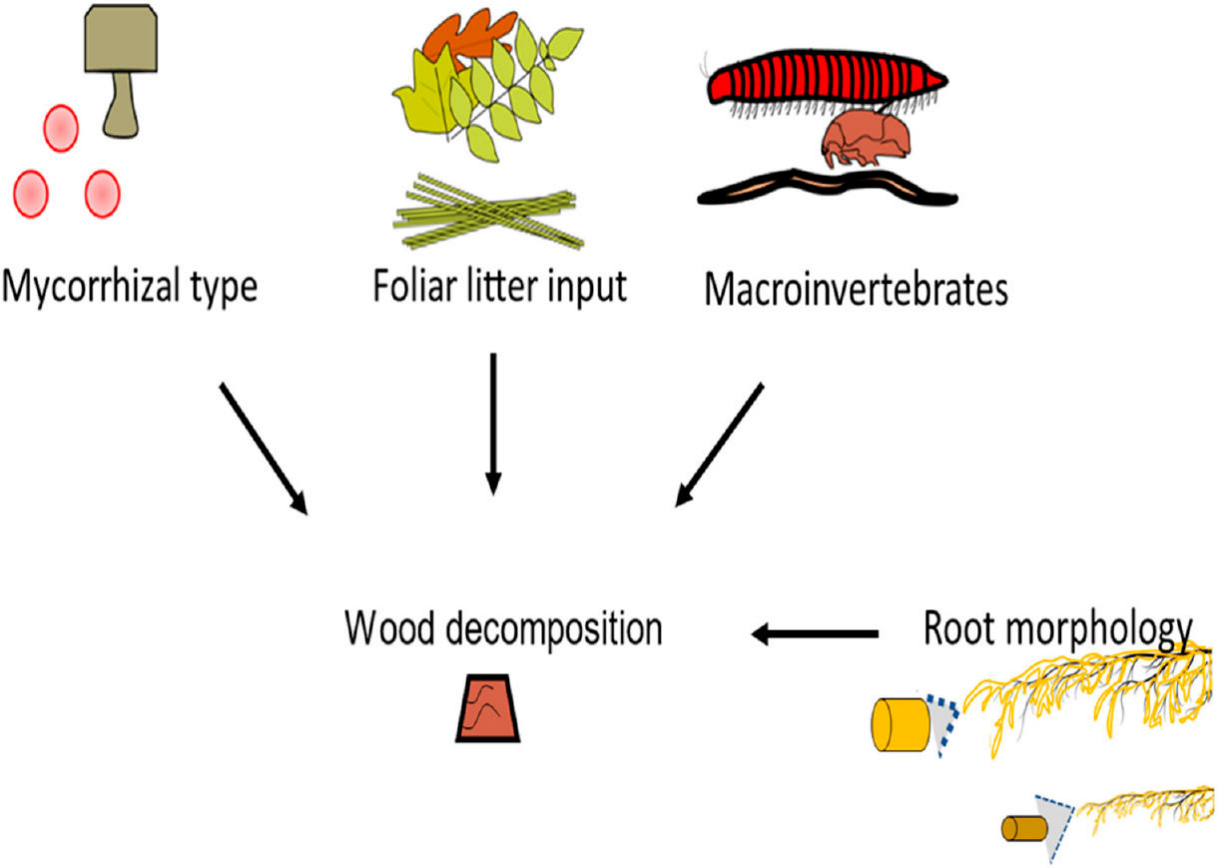
Testing the effects of mycorrhizal type, foliar litter input, root morphology, and macroinvertebrates on wood decomposition in the root zone of 13 temperate trees.

The experiment tested the interactive effect of mycorrhizal type, tree canopy (i.e., foliar traits), root morphology, and macroinvertebrates on decomposition (Figure 1). In addition to selecting a variety of AM and EM trees for this study, the 13 trees were chosen because of their broad root diameter range (Table 1), which was previously determined by assessing roots of the first three orders [54]. As a caveat, DNA and microscopic evidence were not used to verify the mycorrhizal root colonization rate or rare bimodal associations, as this study was designed to only include well-defined mycorrhizal types and tree-specific associations [54,62,63]. The experimental design included 2 conspecific blocks (i.e., 6 trees per conspecific block) × 13 tree species (6 AM + 7 EM) × 2 macroinvertebrates levels for a total of 52 observations. Specifically, this allowed 26 observations that included macroinvertebrates (+M) and 26 observations that excluded macroinvertebrates (−M). In addition, each observation was contrasted by mycorrhizal type, canopy litter trait (needle or broadleaf), and root morphology (roots greater than 0.35 mm (i.e., coarse) versus roots less than 0.35 mm (i.e., fine). The 0.35 mm cut off was chosen because past studies have shown bifurcations in root behavior at this measurement. This includes differences in root length proliferation, root dry mass proliferation, arbuscule mycorrhizal colonization, and extramatrical hyphae length [64].

### 2.4. Wood Substrate

**Figure A1.**
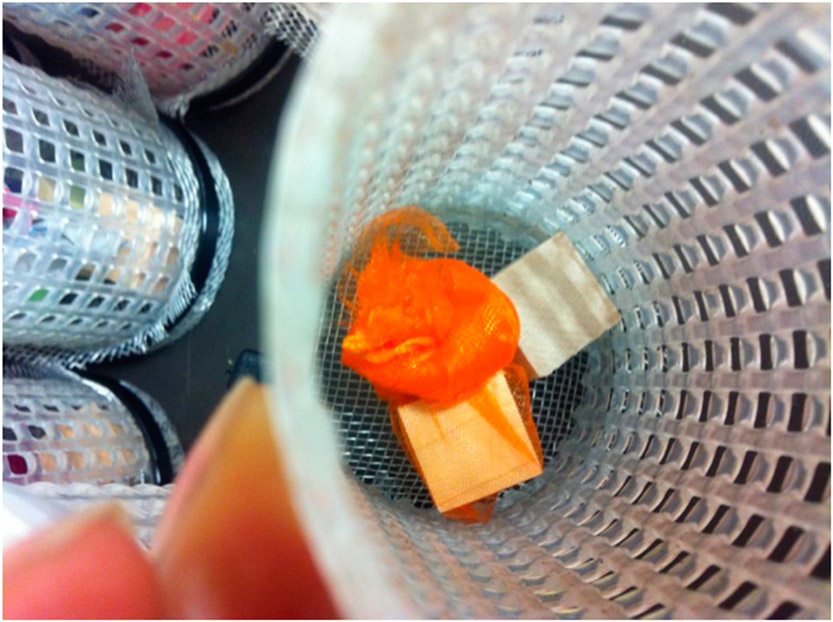
Seen here are cylindrical core mesh tubes with 50 mm × 50 mm window openings. At the base of the cylindrical core are a pair of wood cubes derived from Acer rubrum. The pair is composed of one cube enclosed in a polyester mesh (−M), “while the adjacent pair is void of p olyester mesh + M.

Assessing the rate of wood decomposition for a single species (*Acer rubrum*) was ideal for this study, especially since woody litter can vary in the ratio of labile to recalcitrant tissue [65]. Focusing on a common wood type across diverse tree species also allowed us to examine the influence of tree type and phylogenetic distance on decomposition. Commercial wood cubes with a volume of 1.905 cm^3^ (Woodpeckers Inc., Lakewood, OH, USA), derived from *Acer rubrum*, were oven-dried for 20 h at 40 °C to remove residual moisture in the woody tissue. Wood substrate was handled in pairs (Figure A1), as fine polyester mesh with 0.22 mm openings enclosed one wood substrate (−macroinvertebrates), while another was void of polyester mesh (+macroinvertebrates). Wood pairs were deposited into mesh tube cylindrical cores (10 cm long × 5 cm diameter) with 50 mm × 50 mm openings (Figure A1). These cylindrical cores were found to be unrestrictive to macroinvertebrates and salamanders in previous field studies (i.e., unpublished observations [53,66]).

### 2.5. Field Burial and Incubation

**Figure A2.**
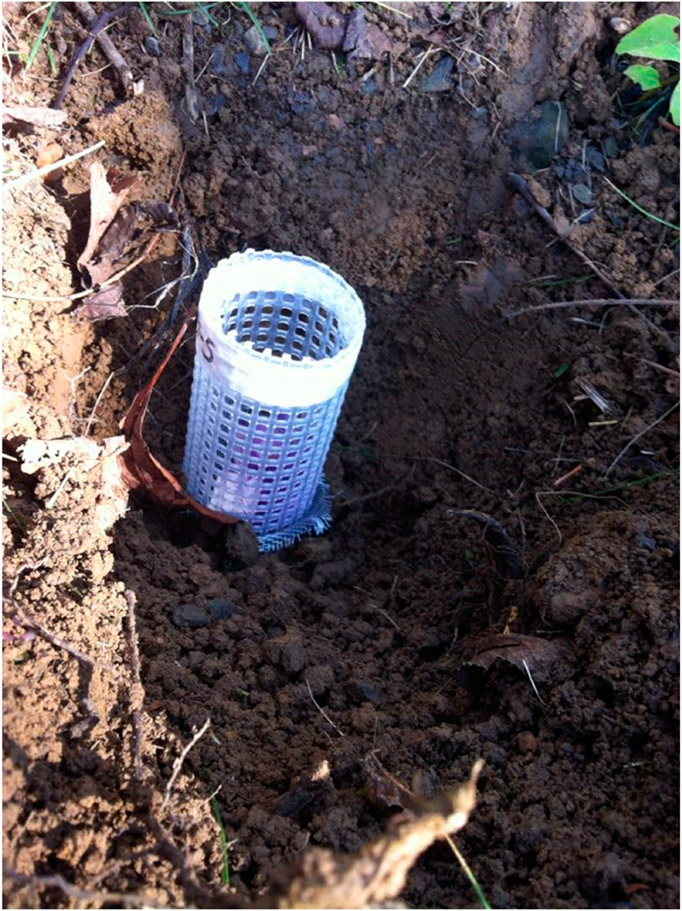
Depicted here is a burial site; cylindrical core was placed vertically into the burial site. The burial and cylindrical core was then filled in with native soil, and wood substrate was allowed to decompose.

Cores were filled and buried vertically (Figure A2) with root-zone soil collected at the burial site. Similar to Malik, Trexler [66], cores were buried ~37.5 cm from the trunk of each specified EM or AM tree. Excavations for core burials were made to about 10 cm, because the decomposition of woody debris frequently occurs at shallow depths [67]. Additionally, cylindrical cores’ subsurface placement enabled wood cubes to stay at a constant depth, as soil surface placement could have potentially led to stochastically uneven burials. Field incubation occurred from October 2017 to November 2018, after which, cores were removed from the field and brought to the laboratory for analysis.

### 2.6. Analysis: Mycorrhizal Type, Foliar Litter, and Macro Invertebrate Exclusion

Wood was oven-dried for 5 days at 40 °C. Decomposition was quantified as proportionate mass loss, because mass loss corresponds to C and N mineralization and lignocellulose solubilization [68,69]. Relative wood decomposition or mass loss was evaluated as the difference in the initial and final mass divided by the initial mass (Delta mass/initial mass). On average, the initial mass was 3 g, and due to the nature of decomposition, the final mass was always less than the initial. This always made relative wood decomposition a positive number between 0 and 1 when plugged into the described formula. Data were analyzed with R version 4.0.2. The Shapiro–Wilk’s test and Levene’s test were used to assess normality and the equality of variance, respectively. Macroinvertebrates, mycorrhizal type, root morphology, and foliar litter trait were held as explanatory variables, while relative wood decomposition was set as the response variable. A four-way ANOVA was performed to determine which explanatory variables were significant predictors at an alpha of 0.05. Statistical outliers were examined with diagnostic plots of standardized residuals and the ‘boxplot () $out’ command. StepAICc was used to evaluate model selection with the ‘drop1()’ command. An association between root diameter and average root zone decomposition was assessed using Pearson product moment correlation through the ‘Hmisc’ package [70], using the ‘rcorr’ command. Correlations between root diameter and average root zone decomposition were further examined with 95% CI ellipses using ggplot2.

### 2.7. Analysis: Phylogenetic Signals

**Figure A3.**
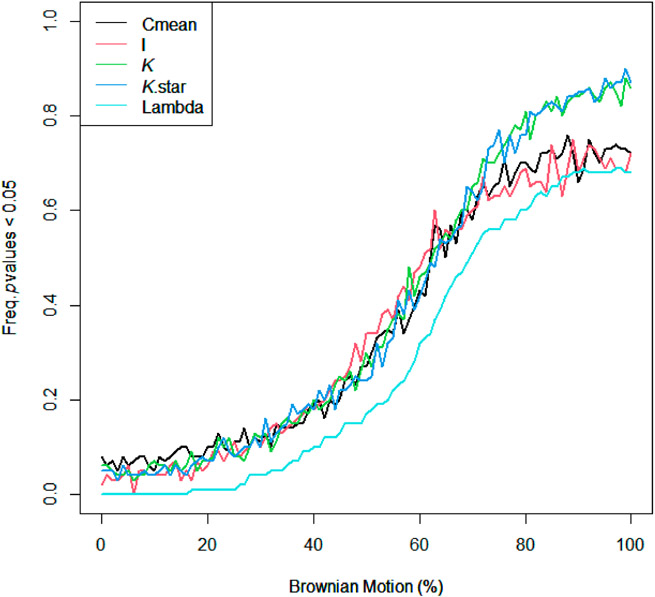
Assessing the behavior of phylogenetic signal via Brownian motion influence gradient.

**Figure A4.**
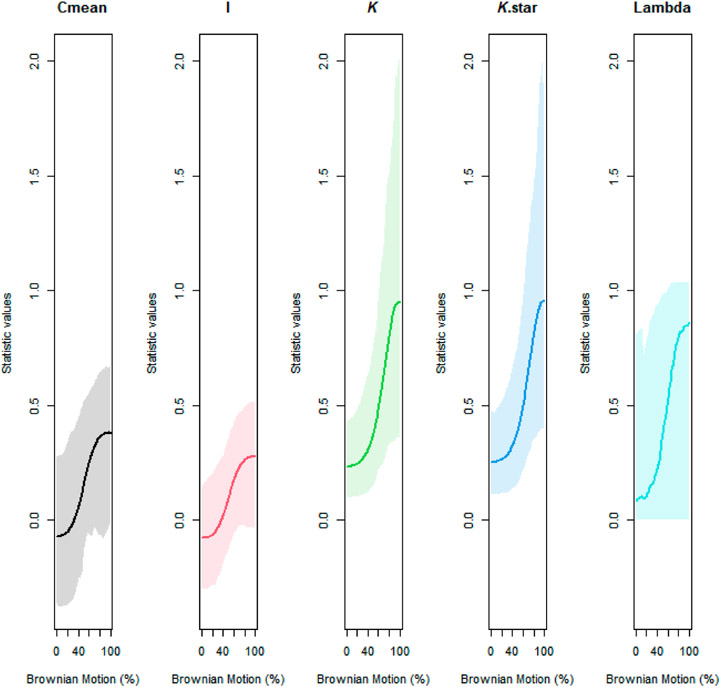
Assessing the behavior of phylogenetic signal via Brownian motion influence gradient and 95% CI.

Using R version 4.1.3, phylogenetic distance was captured with respect to the diverse plant species used in this study. Particularly, ‘*V. Phylomaker*’, an R package with predetermined vascular plant relatedness, was employed [71], and the ‘phytools’ library [72] was used to help generate a phylogenetic construct. Phylosignal for decomposition, root diameter, and Brownian motion (e.g., random effect) was plotted, measured, and tested using the ‘phylosignal’ package [45]. Signal depth was assessed using the ‘phylocorrelogram’ command. Localized signals were then identified using Local indices of phylogenetic association (LIPA), or the ‘lipaMoran’ command, which determined sensitivities and local significance by comparing Local Moran’s index values. The behaviors of general phylogenetic signals were assessed with all methods (Cmean, I, Blomberg’s *K, K*.star, and Pagel’s λ), as seen in Figures A3 and A4, and final statistics were reported with Blomberg’s *K* and Pagel’s λ.

## 3. Results

### 3.1. Phylosignal Associations

**Figure 2.**
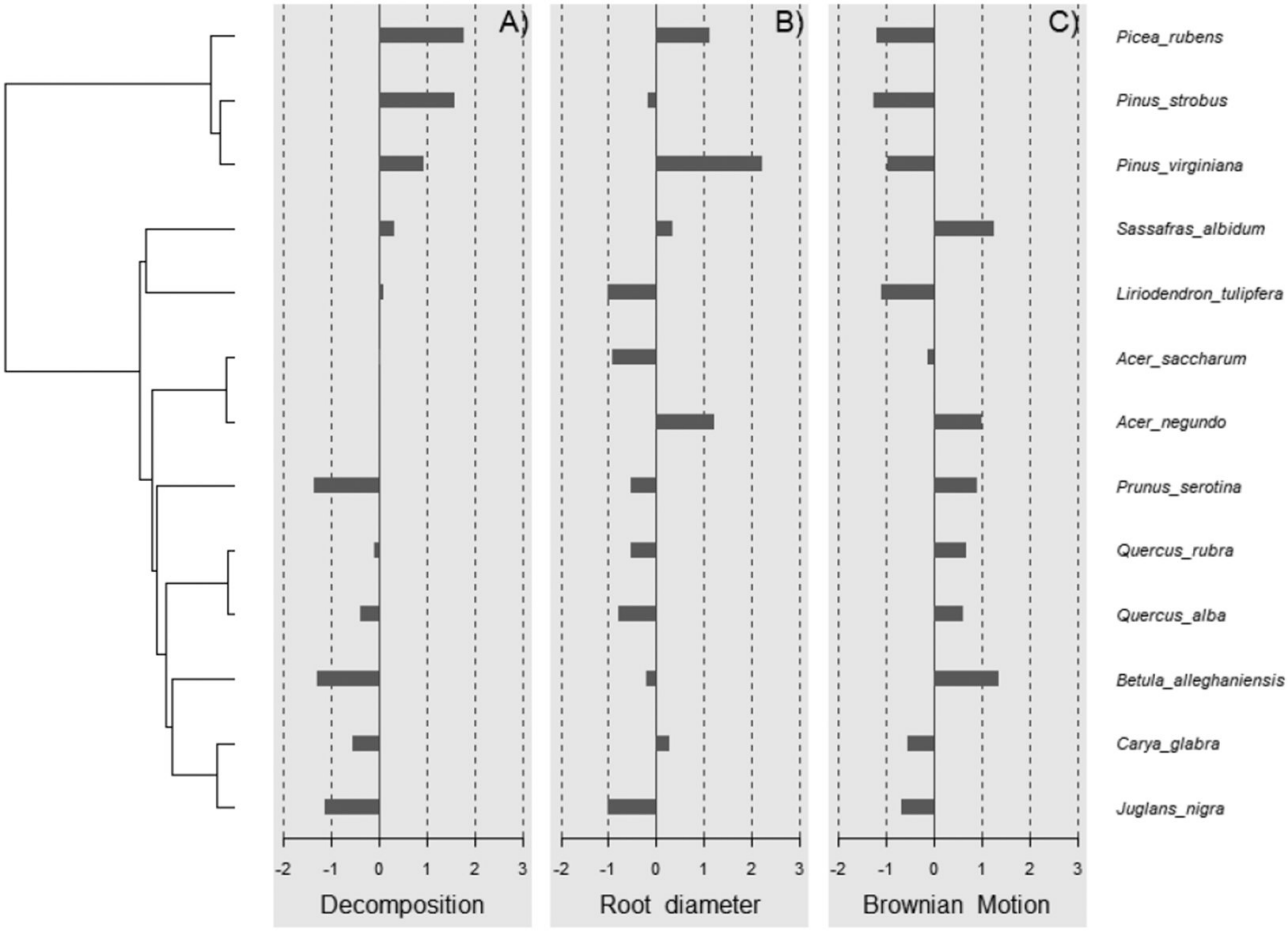
Testing for general phylogenetic signal. A phylogenetic signal detected for tree-soil-mediated wood decomposition (**A**) (Blomberg’s *K* = 1.30, Pagel’s λ = 1.00, *p* = 0.001). The signal was undetected or insignificant for root diameter (**B**) and Brownian motion model (**C**).

**Figure 3.**
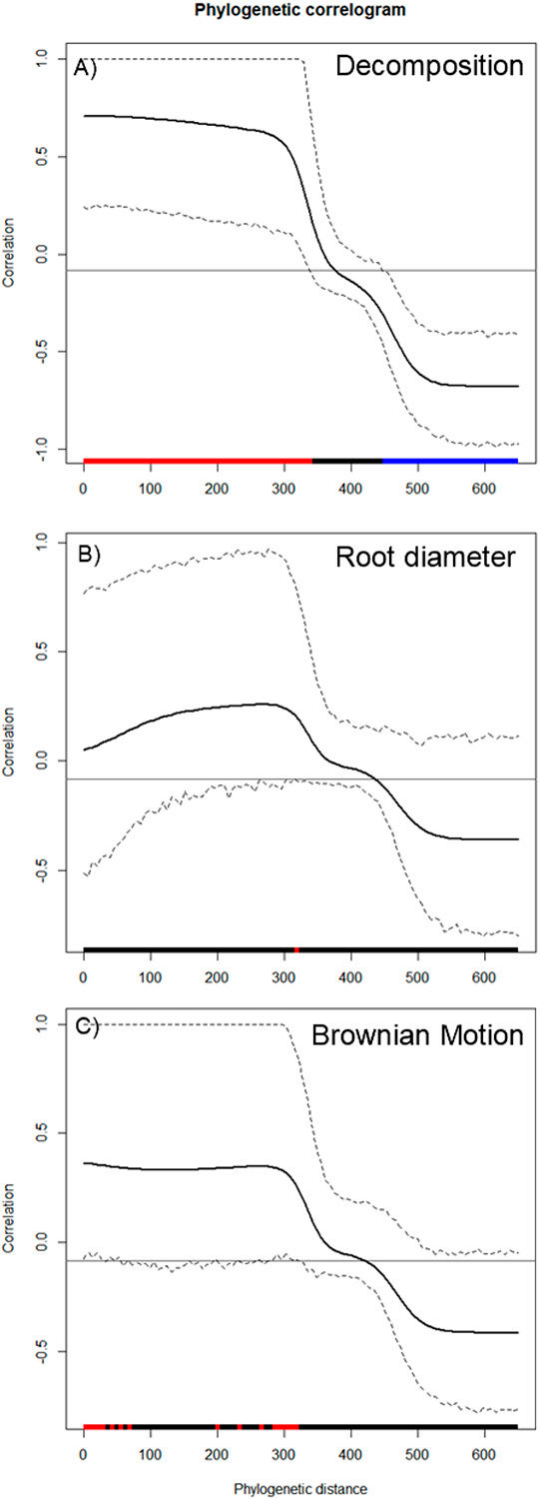
Phylogenetic correlograms for 3 traits: (**A**) decomposition, (**B**) root diameter, and and (**C**) Brownian motion model. Here, correlations (y-axis) are assessed with respect to phylogenetic distance (x-axis). Red bars along the x-axis indicate significant positive autocorrelation, while blue bars along the x-axis indicate significant negative autocorrelation. With respect to the plotted lines, the solid black line represents Moran’s I index of autocorrelation. Hashed lines represent the upper and lower bounds of 95% CI.

**Figure 4.**
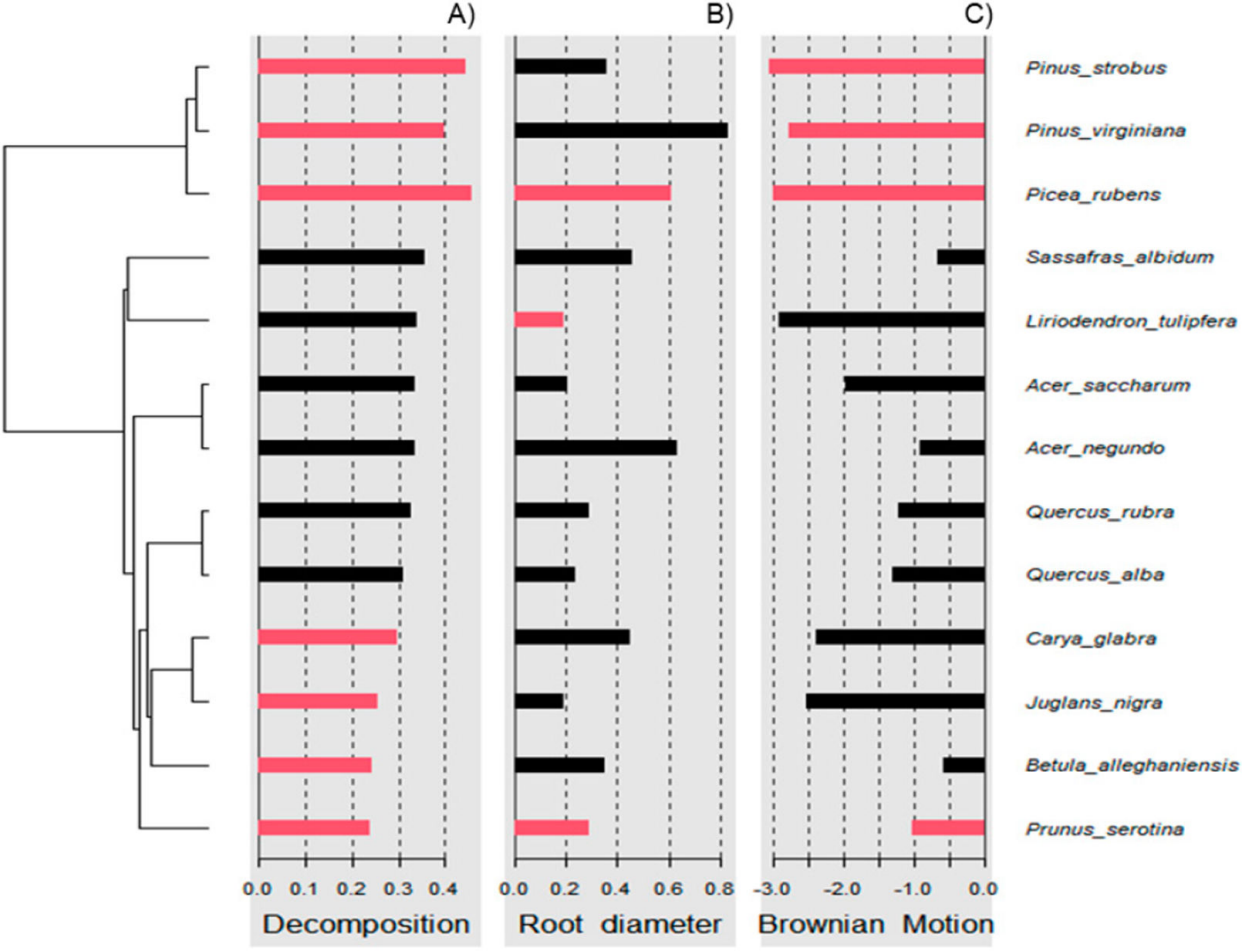
Local indices of phylogenetic association (LIPA) for (**A**) decomposition, (**B**) root diameter, and (**C**) Brownian motion. Red bars indicate significance with respect to Local Moran’s index. As it relates to decomposition, bimodal clustering was observed. Particularly, high sensitivity values were observed for pine species. Meanwhile, low sensitivity values were observed for species distant to pines (i.e., black cherry). With respect to root diameter, sensitivity values were sparse.

Wood decomposition outcomes were associated with a phylogenetic signal (Figure 2A, General tests for phylogenetic signals, Blomberg’s *K* = 1.30, Pagel’s λ = 1.00, *p* = 0.001). The assessment of phylogenetic signal depth via correlograms revealed significant long ranges of positive autocorrelations, and significant long ranges of negative autocorrelation (Figure 3A) for decomposition. However, this was not the case for root diameter and Brownian motion model (Figure 2B,C and Figure 3B,C), as general phylogenetic signals were insignificant for root diameter (Blomberg’s *K* = 0.16, Pagel’s λ = 0.20, *p* = 0.46) and the Brownian motion (Blomberg’s *K* = 0.55, Pagel’s λ = 0.89, *p* = 0.07). With respect to decomposition, Local indices of phylogenetic association (LIPA) revealed bimodal clustering for local Moran’s index values. These clusters were on opposing ends of the phylogenetic spectrum. On one hand, *P. strobus, P. virginiana,* and *P. rubens* had high values of sensitivity (Figure 4A). On the other hand, *Carya, Juglans, Betula,* and *Prunus* species had low values of sensitivity (Figure 4A). Local Moran’s index values were sparse for root diameter (Figure 4B).

### 3.2. Root Diameter, Macroinvertebrates or Mycorrhizae as Drivers

**Figure 5.**
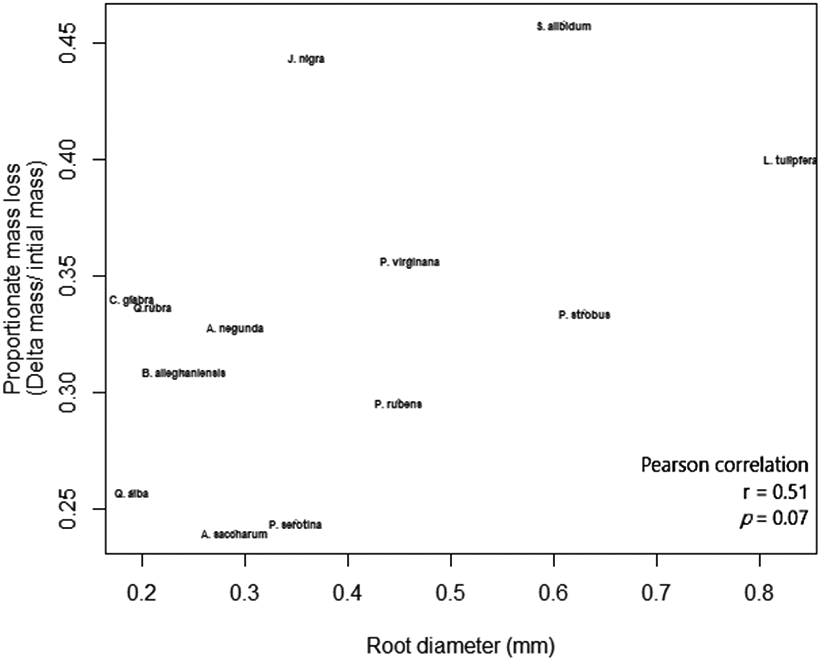
The association between root diameter (mm) and wood decomposition.

**Figure 6.**
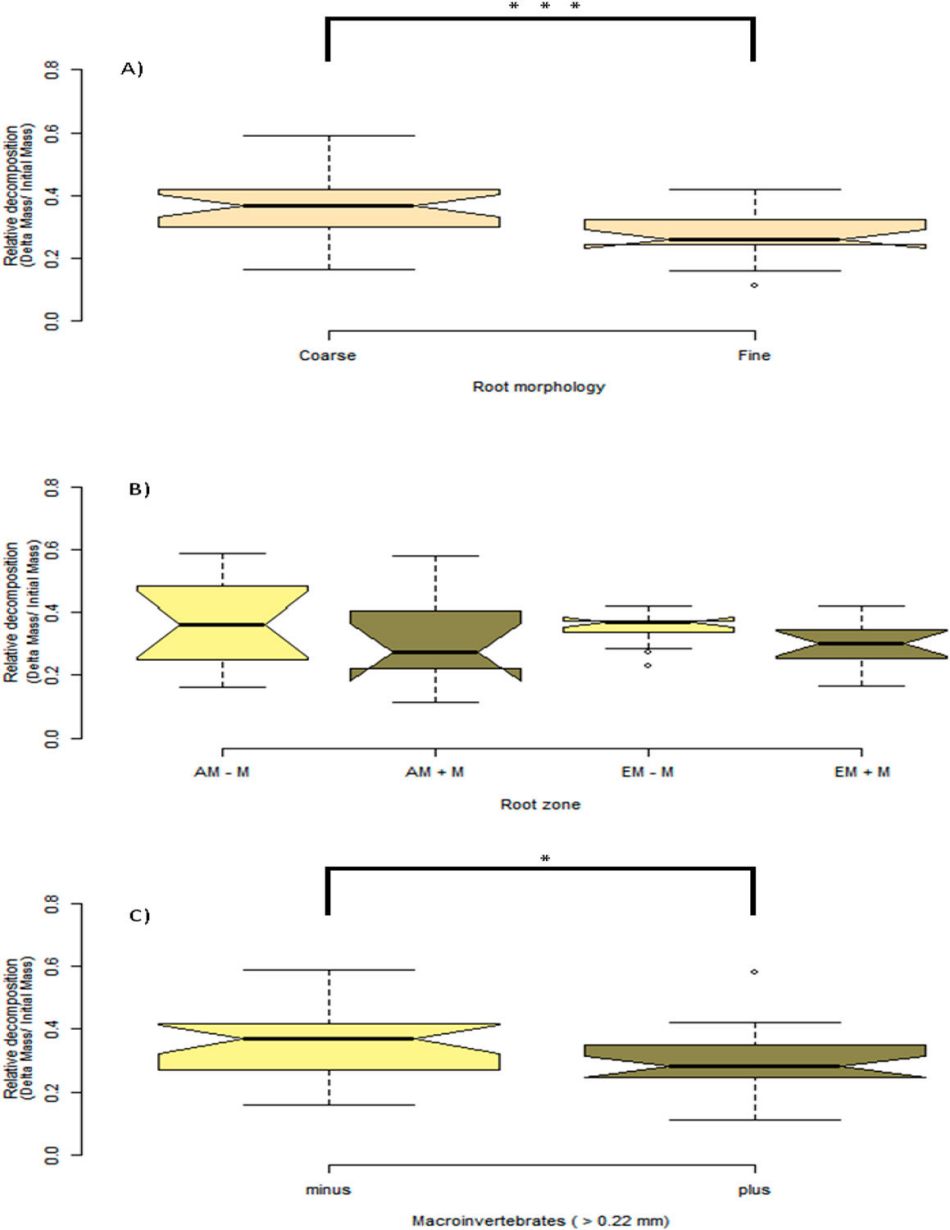
Provided here are boxplots showing the outcome of experimental factors on wood decomposition. The notches in each boxplot represent 95% CI around the mean. The upper edge and lower edge of each boxplot is the 25% and 75% quartile, or the lower and upper median, which are the boundaries of the interquartile range (IQR). In addition, the boxplot whiskers represent 1.5 × IQR, and points beyond the whiskers are outliers. (**A**) Seen here are coarse versus fine root species, and coarse roots are defined as roots > 0.35-mm, and fine roots are defined as < 0.35-mm. (**B**) Root zone and macroinvertebrate effect on decomposition. Light-khaki-colored boxplots represent macroinvertebrate exclusion (−M); dark-khaki-colored boxplots represent non-macroinvertebrate exclusion (+M). (**C**) The main effect of macroinvertebrate exclusion. Significance: * *p* < 0.05; *** *p* < 0.001.

**Figure A5.**
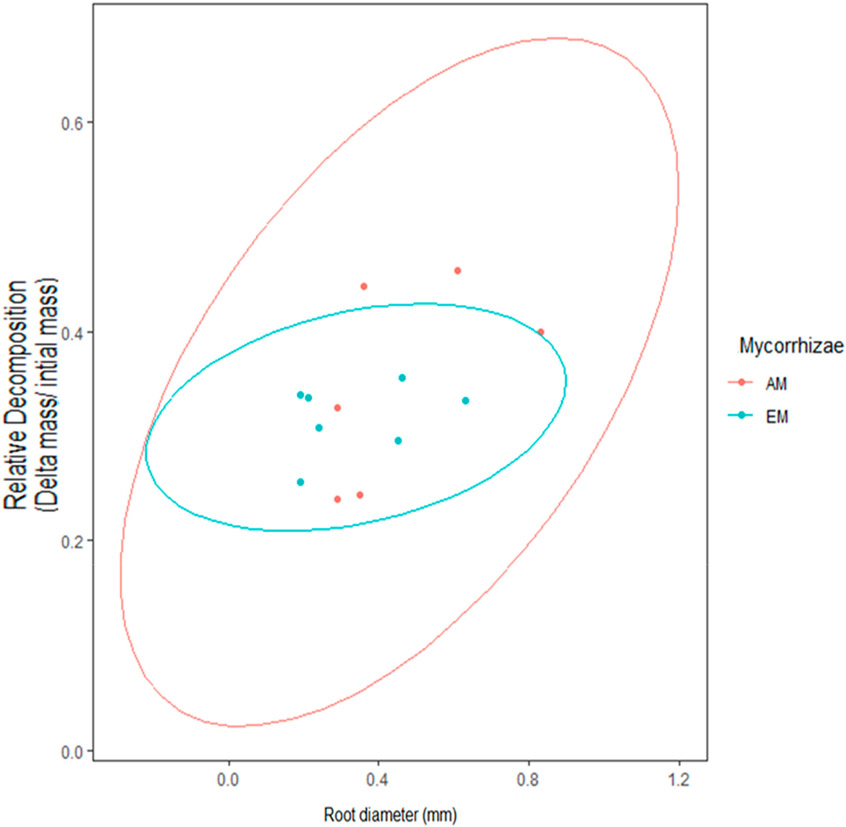
Observed here are 95% CI ellipses in the context of mycorrhizal type. AM root zones had a broader range in decomposition outcomes in comparison to EM root zones.

The outcome of macroinvertebrate, mycorrhizal type, and foliar litter trait on wood decomposition was assessed. Wood decomposition was lowest in the root zone of two AM trees, *A. saccharum* and *P. serotina*, and highest in the root zone of another pair of AM trees, *J. nigra* and *S. albidum* (Figure 5). Foliar litter legacy (broad leaf versus pine needle) did not affect wood decomposition (Table 2), as Step AIC found foliar litter weakened the model. Irrespective of mycorrhizal type, the relationship between root diameter and decomposition was moderately correlated (Figure 5). Specifically, the association between root diameter and wood decomposition was positive and marginally significant (Pearson correlation r = 0.51, *n* = 13, *p* = 0.07). Coarse-rooted species were found to increase decomposition by 8% (Figure 6A, four-way ANOVA, *F*_1,38_ = 0.348, *p* = 0.0004). Statistical overlap/non-overlap between EM and AM root zone outcomes were determined with 95% CI ellipses. With the exception of the AM trees, *J. nigra, S. albidum,* and *L. tulipfera*, there was a great deal of overlap between AM and EM root diameter effects on decomposition (Figure A5). The AM ellipse suggests a positive relationship between root diameter and decomposition, and the EM ellipse suggests a mildly positive relationship (Figure A5). Mycorrhizal type did not significantly influence decomposition (four-way ANOVA, *F*_1,38_ = 0.465, *p* = 0.499), and the difference in decomposition when in AM versus EM root zone was relatively small (i.e., 1.8%). Interestingly, the absence of macroinvertebrates (−macroinvertebrates) increased wood decomposition by about 5.9% (Figure 6C; four-way ANOVA, *F*_1,38_ = 5.10, *p* = 0.029). When only considering AM root zone, −macroinvertebrate was greater than +macroinvertebrate. Similarly, in the EM root zone, −macroinvertebrate was greater than +macroinvertebrate (Figure 6B). No interaction between mycorrhizal type and macroinvertebrates was detected (four-way ANOVA, *F*_1,44_ = 0. 001, *p* = 0.974), but a trend toward significance was observed when examining the interaction between root morphology and mycorrhizal type (four-way ANOVA, *F*_1/38_ = 3.168, *p* = 0.083).

## 4. Discussion

Wood decay provides a sink for N_2_O and a source for CO_2_ and CH_4_ [73], as well as a source for soil phosphorus [65]. In addition, it is widely accepted that wood decomposes faster in soil communities than in suspension [65,73]. The novelty of the present study is the interactive role of root morphology, mycorrhizal type, and macroinvertebrates on wood degradation in rhizosphere-adjacent soils. Soils are differentially influenced by plantspecific traits (i.e., rhizodeposition and foliage), which in turn helps structure belowground communities [74]. To our knowledge, this is the first study to show an association between phylogenetic distance and wood decomposition. Species that were more closely related to pines had highly sensitive values for local positive auto correlations. Tree mycorrhizal type also has the potential to impact root zone dynamics. Past studies have predicted differences in AM and EM trees in biogeochemical cycles [63,75,76], which may be pertinent to shifts in forest demography [77], which can impact predominant mycorrhizal type [78]. As it relates to this study, mycorrhizal type did not impact wood decomposition (Table 2), which suggests a mixed effect of mycorrhizal type on C cycling that may depend on the species of fungi colonizing the host tree species. To our knowledge, we are the first to examine wood decomposition in root zones of contrasting mycorrhizal association and root morphology. Mild interaction was observed between mycorrhizal type and root diameter (Table 2). Irrespective of mycorrhizal type, root diameter was positively correlated with wood decomposition at a marginal level of significance (Figure 5), as coarse roots significantly increased decomposition (Figure 6A). While coarse roots promoted decomposition, a phylogenetic signal was not detected for this trait. Interestingly, excluding macroinvertebrates from the soil environment led to increased wood degradation (Figure 6C), which corroborates the findings of Wood, Tordoff [20], which suggested that macroinvertebrates can disrupt microbial decomposer involvement in wood decomposition. Taken together, these findings provide insight into wood decomposition as influenced by biodiverse root zones.

### 4.1. Foliar Trait Legacy and Wood Decomposition

Phylogenetic signals were observed in tree-specific soils (Figure 2). These signals were likely due to root exudates and litter input from the canopy trees. Litter inputs can fuel soil food webs through rhizodeposits, foliage, and surface accumulation [79]. Specifically, plant identity can structure communities of arachnida, nematoda, collembola, enchytraeidae, and mycorrhizae [74]. Together, these communities may help facilitate the outcome of wood decomposition. Lignin is an important component of wood and makes up 20–32% of lignocellulosic biomass and is dramatically resistant to chemical degradation [33,80]. According to our ANOVA model, the decomposition rate beneath pine versus broad leaf canopies was insignificant (Table 2), but these differences may be gradual and amplified over phylogenetic distance (Figures 2A and 3A). This may also explain why local positive autocorrelation was detected on opposite ends of the phylogenetic spectrum (Figure 4A). Sensitivity values waned in species that were phylogenetically distant from *Pinus* and *Picea* species, including *Carya* and *Juglans* spp. Ironically, *Carya glabra* and *Juglans nigra* were previously shown to differ from *Pinus strobus* and *Pinus virginiana* in stem wood density [81] by about 45 percent. The tree species used in this study have been structuring the soil community for over 20 years at this particular site, yielding notable changes in some soil characteristics [61]. The effect of tree litter deposits may amplify with an increase in stand age [82], as tree-specific litter has been reported to impact invertebrate richness, diversity, and assemblage [24]. The phylogenetic signal observed in the present study suggests tree species can condition the soil environment in way that may be predictable across species of closely related genera.

### 4.2. Macroinvertebrate Exclusion Improved Wood Decomposition

Macroinvertebrate exclusion increased wood decomposition (Table 2). This may suggest that macroinvertebrates can disrupt saprotroph mycelial cords and reduce C cycling. On the contrary, there may be a specific context in which macroinvertebrates and fungal decomposers can have an additive effect on wood decomposition, and this may depend on the abundance of macroinvertebrates that specialize in wood (i.e., termites, carpenter ants, etc.). For example, +macroinvertebrate communities that are overrepresented with termites and carpenter ants are likely to increase wood decomposition, as this select class of arthropods can specialize in recalcitrant forms of carbon. However, this experiment was conducted in the root zone, which is rich in labile carbon (i.e., active photosynthates, soluble C, etc.) and root-derived C [83], thereby providing resources to a broad array of organisms. Macroinvertebrate exclusion improved subsurface wood decomposition (Figure 6C), and similar effects have been found for surface wood decomposition, as macroinvertebrate exclusion was reported to alter saprotroph communities [16]. In subtropical forests, wood contact with soil surface, combined with invertebrates, was found to be optimal for wood decomposition [84]. The role of macroinvertebrates may also depend on canopy tree cover (i.e., shading); an increase in ambient radiation has been shown to decrease invertebrate density [47].

### 4.3. Mycorrhizal Type and Wood Decomposition

Mycorrhizal type has important implications on ecosystem processes, including soil structure, C storage, and N and P cycling [85-87]. As it relates to global change biology, predictable demographic shifts in eastern USA deciduous forests [77] can directly lead to shifts in the dominant mycorrhizal type. Mycorrhizal fungi can increase the uptake of soluble nutrients, and in some cases, the mycorrhizal type (e.g., EM fungi) can even lead to the decomposition of organic matter [37]. However, this may depend on the forest system (i.e., boreal versus temperate forest), as EM’s lignocellulolytic enzyme capabilities may not necessarily apply to recalcitrant woody litter, although EM fungi have been found to modify SOM [88,89]. Mycorrhizal type did not influence wood decomposition in this study (Table 2), perhaps suggesting that models predicting the role of mycorrhizae in C and nutrient cycling may not apply to wood-derived C in temperate forests.

### 4.4. Moderate Correlation between Decomposition and Root Diameter

Our findings suggest wood decomposition may not be impacted by tree demographic shifts affecting mycorrhizal type, but instead, by tree-species shifts affecting absorptive root diameter. Across the root zone of 13 diverse tree species, we found a positive correlation between root diameter and wood decomposition (Figure 5), with most points falling within the 95% CI (Figure A5). Wood decomposition in coarse root zone soils was increased by ~8% (*p* < 0.001; Figure 6A). Perhaps this may be explained by the “drying effect hypothesis” [42], where fine roots are better able to remove soil residual moisture from soil and suppress decomposer activity. In grasses, root diameter was found to be positively correlated with decomposition and soil organic carbon respiration [43]. Interestingly, a similar trend was found among woody plants [44], perhaps suggesting that increased root diameter facilitates increased C and N cycling. Collectively, these findings show an additive effect of root diameter on decomposition. In addition, we did not observe interaction of root diameter with the macroinvertebrate exclusion barrier (0.22 mm mesh). This suggests that while the barrier may have inhibited a certain amount of root growth around the wood substrate, it did not limit the growth of coarse-root species more than fine-root species. To our knowledge, this is the first study to show a correlation of root diameter with the decomposition of wood debris.

## 5. Conclusions

Unraveling ways in which biodiverse trees can influence ecosystem processes may provide additional insight into C and nutrient cycling. With respect to the root zone, a phylogenetic signal was observed for decomposition. *Pinus* and *Picea* species were most sensitive to decomposition–phylogenetic distance autocorrelations. The presence of macroinvertebrates lessened recalcitrant litter decomposition, perhaps suggesting an antagonistic effect of macroinvertebrates on saprotrophs. Additionally, the finding that mycorrhizal type did not affect wood decomposition suggests neutral outcomes for forest demography shifts, specifically those that affect predominant mycorrhizal type (i.e., AM versus EM). Additionally, results of this study support the premise that woodlands overrepresented by trees of a large root diameter can potentially accelerate the cycling of recalcitrant C.

## Figures and Tables

**Table 1.** Root diameter, mycorrhizal type, and foliar litter trait from 13 temperate trees at a plantation in Pennsylvania, USA.

Mycorrhizal Type	Species	Common Name	Canopy/Foliar Litter	Root Diameter (mm)
AM	*Acer negundo*	Box elder	Broad leaf	0.29
	*Acer saccharum*	Sugar maple	Broad leaf	0.29
	*Prunus serotina*	Black cherry	Broad leaf	0.35
	*Juglans nigra*	Eastern American black walnut	Broad leaf	0.36
	*Sassafras albidum*	Sassafras	Broad leaf	0.61
	*Liriodendron tulipfera*	Tulip poplar	Broad leaf	0.83
EM	*Carya glabra*	Pignut hickory	Broad leaf	0.19
	*Quercus alba*	White oak	Broad leaf	0.19
	*Quercus rubra*	Northern red oak	Broad leaf	0.21
	*Betula alleghaniensis*	Yellow birch	Broad leaf	0.24
	*Picea rubens*	Red spruce	pine	0.45
	*Pinus virginiana*	Virginia pine	pine	0.46
	*Pinus strobus*	White pine	pine	0.63

Root diameter at this site was previously described by Chen et al. (2016) [54]. Diameter is an estimate of roots of the first three orders.

**Table 2.** Four-way ANOVA.

Source of Wood Decomposition	Df	Sum Sq	Mean Sq	F Value	Pr (>F)
**Macroinvertebrates**	**1**	**0.0429**	**0.0429**	**5.1010**	**0.0297** *
Mycorrhizal type	1	0.0039	0.0039	0.4651	0.4993
Foliar litter	1	0.0045	0.0045	0.5431	0.4656
**Root morphology**	**1**	**0.1250**	**0.1249**	**14.842**	**0.0004** ***
Macroinvertebrate: Mycorrhizal type	1	0.0000	0.0000	0.0010	0.9747
Macroinvertebrate: Foliar litter	1	0.0021	0.0021	0.2546	0.6147
Macroinvertebrates: Root morph	1	0.0007	0.0007	0.0855	0.7715
Mycorrhizal type: Root morph	1	0.0266	0.0266	3.1682	0.0830
Macroinvertebrates: Mycorrhizae: Root morph	1	0.0000	0.0000	0.0014	0.9705
Residuals	38	0.3200	0.0084		

Four-way ANOVA: assessing the effect of experimental predictors. Asterisks and bold font indicate significance. Note: Root morphology is a comparison of coarse versus fine root species; coarse roots > 0.35 mm and fine roots < 0.35 mm. Significance:

* *p* < 0.05

*** *p* < 0.001

## Data Availability

Data is to be made available upon request.
